# Creatinine-cystatin C ratio and death with a functioning graft in kidney transplant recipients

**DOI:** 10.1038/s41598-024-52649-5

**Published:** 2024-01-23

**Authors:** Mun Chae Choi, Deok Gie Kim, Seung Hyuk Yim, Hyun Jeong Kim, Hyoung Woo Kim, Jaeseok Yang, Beom Seok Kim, Kyu Ha Huh, Myoung Soo Kim, Juhan Lee

**Affiliations:** 1 Department of Surgery, Yonsei University College of Medicine, Seoul, Republic of Korea; 2 The Research Institute for Transplantation, Yonsei University College of Medicine, Seoul, Republic of Korea; 3 Department of Internal Medicine, Yonsei University College of Medicine, Seoul, Republic of Korea

**Keywords:** Allotransplantation, Chronic kidney disease, Biomarkers, Outcomes research

## Abstract

Death with a functioning graft is important cause of graft loss after kidney transplantation. However, little is known about factors predicting death with a functioning graft among kidney transplant recipients. In this study, we evaluated the association between post-transplant creatinine-cystatin C ratio and death with a functioning graft in 1592 kidney transplant recipients. We divided the patients into tertiles based on sex-specific creatinine-cystatin C ratio. Among the 1592 recipients, 39.5% were female, and 86.1% underwent living-donor kidney transplantation. The cut-off value for the lowest creatinine-cystatin C ratio tertile was 0.86 in males and 0.73 in females. The lowest tertile had a significantly lower 5-year patient survival rate and was independently associated with death with a functioning graft (adjusted hazard ratio 2.574, 95% confidence interval 1.339–4.950, *P* < 0.001). Infection was the most common cause of death in the lowest tertile group, accounting for 62% of deaths. A low creatinine-cystatin C ratio was significantly associated with an increased risk of death with a functioning graft after kidney transplantation.

## Introduction

Kidney transplantation (KT) is the preferred treatment for patients with end-stage kidney disease. Although short-term outcomes of KT have continued to improve over the past couple of decades, long-term outcomes remain suboptimal^1,2^. Previous studies on long-term outcomes post-KT have focused on death-censored graft failure^3,4^. Graft outcomes have improved in recent years through evolutions in clinical practice, including accurate measurement of graft renal function, immunologic monitoring, and histologic evaluation^5–7^. However, death with a functioning graft (DWFG)—an important cause of overall graft loss—has received little attention^8–10^. Given that the number of elderly KT recipients with multiple comorbidities continues to rise, DWFG is becoming an increasingly relevant issue^9,10^. Therefore, clinical parameters identifying patients at higher risk for DWFG are urgently required.

Serum concentration of cystatin C, a cysteine protease inhibitor produced by all nucleated cells, has been proposed as a measure that may be superior to creatinine as a proxy for glomerular filtration of the kidneys^11^. Interestingly, evidence suggests that cystatin C is linked to mortality risk independently of renal function^12,13^. Furthermore, several studies have investigated the serum creatinine-cystatin C ratio as a surrogate marker for mortality^14,15^. This ratio is easy to obtain and minimizes the influence on renal function. Nevertheless, the clinical relevance of serum creatinine-cystatin C ratio in KT recipients has not been heretofore evaluated.

In the present study, we investigated the association between post-transplantation creatinine-cystatin C ratio and DWFG in a large KT cohort.

## Results

### Baseline characteristics

Among the 1592 recipients included in this study, 39.5% were female, and 86.1% underwent living-donor KT. Donor, recipient, and transplantation characteristics are summarized in Table 1. The mean age of recipients at the time of KT was 42.8 years, and their mean body mass index (BMI) was 22.4 kg/m^2^. Recipients in the lowest tertile received dialysis for a longer time before KT, were more likely to be underwent re-transplantation, and were more likely to be older at the time of KT. There were also significant differences in donor age and donor sex between creatinine-cystatin C ratio tertiles.

**Table 1:** Donor, recipient, and transplantation characteristics according to creatinine-cystatin C ratio tertile.

Variables	Lowest tertile (n = 530)	Middle tertile (n = 541)	Highest tertile (n = 521)	*P*
Recipient age, y*	46.2 ± 10.9	43.3 ± 11.1	38.9 ± 10.5	< 0.001
Sex, female	209 (39.4%)	210 (38.8%)	210 (40.3%)	0.883
BMI, kg/m^2^	22.1 [20.0, 23.9]	22.1 [20.2, 24.3]	22.4 [20.3, 24.3]	0.381
BMI > 25 kg/m^2^	80 (17.0%)	85 (18.5%)	78 (17.1%)	0.789
Re-transplant	74 (14.0%)	46 (8.5%)	28 (5.4%)	< 0.001
HLA mismatch	3 [2, 3]	3 [2, 4]	3 [2, 4]	0.162
Duration of dialysis, mo	9 [1, 48]	8 [1, 34]	4 [1, 25]	< 0.001
Deceased donor	80 (15.1%)	79 (14.6%)	63 (12.1%)	0.322
Donor age, y*	38.3 ± 11.5	39.8 ± 11.9	42.3 ± 11.3	< 0.001
Donor sex, female	219 (41.3%)	255 (47.1%)	268 (51.4%)	0.004

Data are presented as number (percentage), mean ± standard deviation, or median [interquartile range].

BMI, body mass index; HLA, human leukocyte antigen.

*At the time of transplantation.

Baseline patient characteristics at the time of creatinine-cystatin C ratio measurement are shown in Table 2. Overall, the median time from transplantation to ratio measurement was 121 months (IQR, 24–196 months). Recipients in the lowest creatinine-cystatin C ratio tertile were significantly older and had a higher prevalence of diabetes mellitus and hypertension. The majority of recipients received calcineurin inhibitors (98.7%) in combination with a glucocorticoid (83.7%) and antimetabolite (75.8%) after KT. Cyclosporin was used more frequently in the lowest tertile, whereas tacrolimus and an antimetabolite were used more frequently in the highest tertile. The median serum creatinine increased (first to third tertile: 1.07, 1.18, and 1.36 mg/dL, respectively) and the median cystatin C level decreased (first to third tertile: 1.47, 1.32, and 1.29 mg/L, respectively) as the creatinine-cystatin C ratio increased. The lowest creatinine-cystatin C ratio tertile had significantly lower hemoglobin, albumin, total cholesterol, and uric acid, compared with the other tertiles.

**Table 2:** Baseline characteristics at the time of creatinine-cystatin C ratio measurement according to creatinine-cystatin C ratio tertile.

Variables	Lowest tertile (n = 530)	Middle tertile (n = 541)	Highest tertile (n = 521)	*P*
Recipient age, y	58.7 ± 10.1	53.4 ± 9.8	46.7 ± 10.7	< 0.001
Time after transplantation, mo	155 [69, 232]	122 [25, 198]	85 [12, 153]	< 0.001
Hemoglobin, g/dL	13.0 [11.6, 14.3]	13.3 [12.0, 14.5]	13.2 [11.8, 14.4]	0.027
Albumin, g/dL	4.1 [3.9, 4.3]	4.3 [4.1, 4.4]	4.3 [4.1, 4.5]	< 0.001
BUN, mg/dL	20.2 [16.0, 29.2]	20.6 [16.6, 26.6]	21.1 [16.4, 30.2]	0.233
Creatinine, mg/dL	1.07 [0.84, 1.35]	1.18 [0.97, 1.46]	1.36 [1.14, 1.92]	< 0.001
eGFR, mL/min/1.732 m^2^	57.9 ± 21.8	60.4 ± 20.7	55.9 ± 24.8	0.005
Cystatin C, mg/L	1.47 [1.20, 1.95]	1.32 [1.12, 1.63]	1.29 [1.08, 1.77]	< 0.001
Calcium, mg/dL	9.2 [8.9, 9.6]	9.3 [9.0, 9.6]	9.3 [8.9, 9.6]	0.207
Total cholesterol, mg/dL	162 [141, 189]	169 [148, 194]	175 [154, 201]	< 0.001
Phosphorus, mg/dL	3.4 [3.0, 3.8]	3.4 [3.0, 3.8]	3.4 [3.0, 3.8]	0.276
Uric acid, mg/dL	5.0 [4.1, 6.5]	5.0 [4.2, 6.3]	5.6 [4.5, 6.7]	< 0.001
tCO_2_, mmol/L	26.0 [24.0, 29.0]	26.0 [24.0, 28.0]	25.0 [23.0, 28.0]	< 0.001
Maintenance immunosuppressants				
Cyclosporine	151 (28.5%)	102 (18.9%)	87 (16.7%)	< 0.001
Tacrolimus	374 (70.6%)	430 (79.5%)	428 (82.1%)	< 0.001
Glucocorticoid	426 (80.4%)	457 (84.5%)	450 (86.4%)	0.026
MMF	271 (51.1%)	329 (60.8%)	323 (62.0%)	< 0.001
mTORi	61 (11.5%)	49 (9.1%)	51 (9.8%)	0.394
Diabetes mellitus	237 (44.7%)	192 (33.5%)	144 (27.6%)	< 0.001
Hypertension	417 (78.7%)	382 (70.6%)	382 (73.3%)	0.009

Data are presented as number (percentage), mean ± standard deviation, or median [interquartile range].

BUN, blood urea nitrogen; eGFR, estimated glomerular filtration rate; MMF, mycophenolate mofetil; mTORi, mammalian target of rapamycin inhibitor; tCO_2_, total carbon dioxide.

### Creatinine-cystatin C ratio

**Figure 1:**
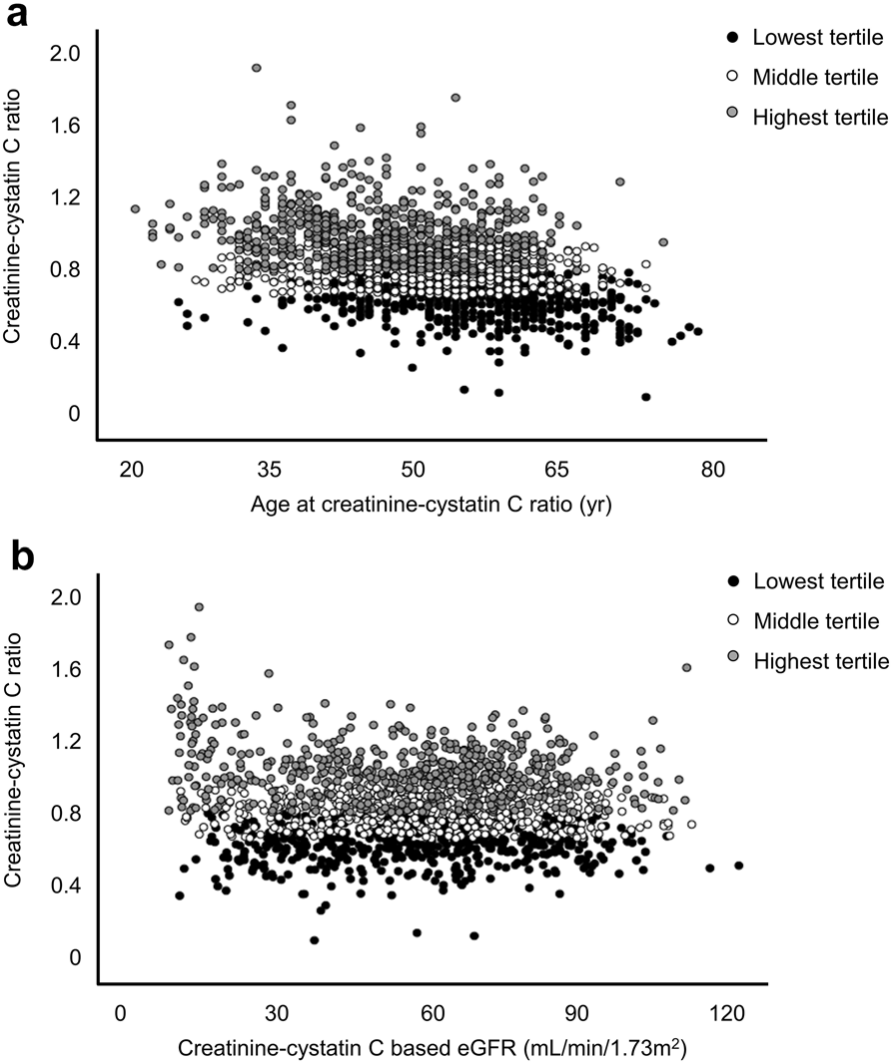
Distributions of the creatinine-cystatin C ratio and (**A**) age and (**B**) creatinine-cystatin C based eGFR. eGFR, estimated glomerular filtration rate.

There was a strong positive correlation between serum creatinine and cystatin C levels (males: r = 0.850, *P* < 0.001; females: r = 0.871, *P* < 0.001) (Fig. S1). The cut-off value for the lowest creatinine-cystatin C ratio tertile was 0.86 in males and 0.73 in females. Distributions of the creatinine-cystatin C ratio and the age and creatinine-cystatin C based eGFR are shown in Fig. 1.

To investigate risk factors associated with a low creatinine-cystatin C ratio, we performed multivariable logistic regression analysis using the lowest tertile as a binary, dependent variable. In multivariable analysis, the following were independently associated with the lowest creatinine-cystatin C ratio tertile: older recipient age at the time of creatinine-cystatin C ratio measurement (adjusted odds ratio [aOR] 1.082, 95% confidence interval [CI] 1.069–1.095; *P* < 0.001), re-transplant (aOR 2.215, 95% CI 1.526–3.214; *P* < 0.001), and longer duration of dialysis (aOR 1.005, 95% CI 1.002–1.007; *P* = 0.001) (Table 3).

**Table 3:** Risk factors for low creatinine-cystatin C ratio (logistic regression analysis).

Variables	Univariable		Multivariable^a^	
	OR (95% CI)	*P*	aOR (95% CI)	*P*
Recipient age*	1.083 (1.070, 1.095)	< 0.001	1.082 (1.069, 1.095)	< 0.001
Diabetes mellitus	1.748 (1.410, 2.166)	< 0.001	1.227 (0.967, 1.557)	0.093
Re-transplant	2.167 (1.541, 3.046)	< 0.001	2.215 (1.526, 3.214)	< 0.001
Dialysis duration	1.004 (1.002, 1.006)	0.001	1.005 (1.002, 1.007)	0.001
BMI*	0.984 (0.950, 1.019)	0.367		
Deceased donor transplant	1.152 (0.856, 1.549)	0.350		
eGFR ≥ 90 mL/min/1.732 m^2^	Reference			
60 ≤ eGFR < 90 mL/min/1.732 m^2^	0.870 (0.574, 1.320)	0.514		
30 ≤ eGFR < 60 mL/min/1.732 m^2^	1.045 (0.688, 1.588)	0.837		
eGFR < 30 mL/min/1.732 m^2^	0.819 (0.504, 1.331)	0.420		
Glucocorticoid use	0.700 (0.532, 0.920)	0.011	0.943 (0.697, 1.278)	0.707

^a^Model was established using the backward conditional method, entering covariates with *P* < 0.2 on univariable analysis.

*At the time of creatinine-cystatin C ratio measurement.

aOR, adjusted odds ratio; BMI, body mass index; CI, confidence interval; eGFR, estimated glomerular filtration rate; OR, odds ratio.

### Death with a functioning graft

**Figure 2:**
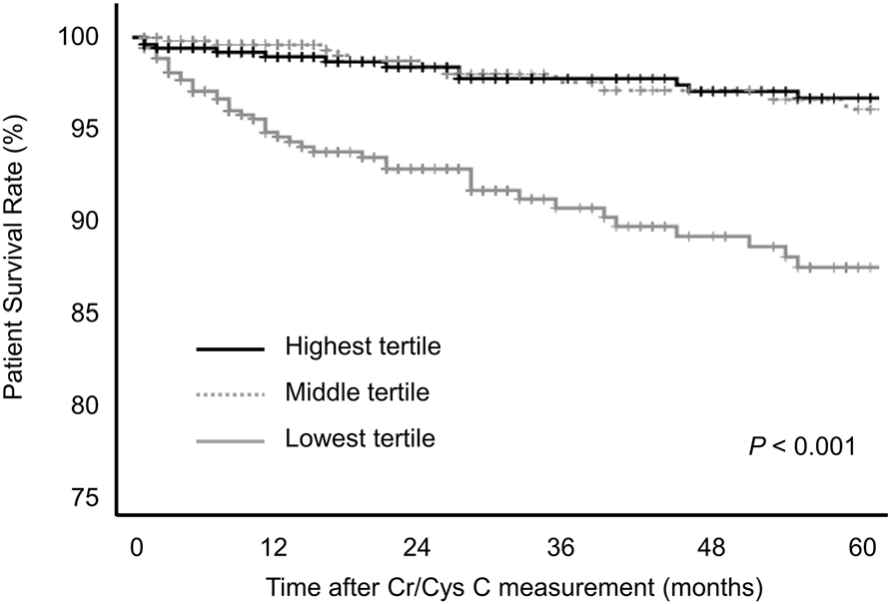
The Kaplan–Meier curve for patient survival in creatinine-cystatin C ratio groups.

**Figure 3:**
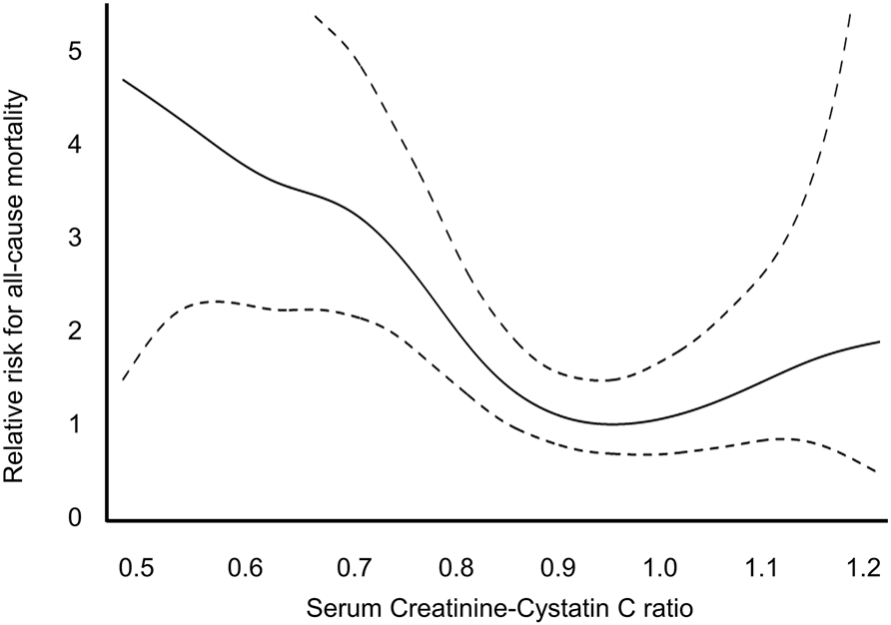
Cubic spline model about relationship between serum creatinine-cystatin C ratio and all-cause mortality.

The median duration of follow-up after creatinine-cystatin C ratio measurement was 28 months (IQR, 11.0–87.0 months). During follow-up, DWFG occurred in 78 patients. There was a significant difference in patient survival between creatinine-cystatin C ratio groups. The 5-year patient survival rate after creatinine-cystatin C measurement was 87.3% in the lowest tertile, 96.0% in the middle tertile, and 96.7% in the highest tertile (*P* < 0.001) (Fig. 2). In multivariable Cox proportional hazard analysis, the lowest creatinine-cystatin C ratio tertile was significantly associated with DWFG (adjusted hazard ratio [aHR] 2.574, 95% CI 1.339–4.950; *P* = 0.005) (Table 4, model 3). This association was further evaluated using cubic spline analysis (Fig. 3). In the competing risk analysis, the lowest tertile group showed a significant increase in the hazard for DWFG, considering the competing risk of death-censored graft failure (HR 1.478, p < 0.001) (Fig. S2).

**Table 4:** Risk factors for death with a functioning graft according to creatinine-cystatin C ratio tertile (Cox proportional hazard regression models).

	Model 1		Model 2		Model 3	
	HR (95% CI)	*P*	HR (95% CI)	*P*	HR (95% CI)	*P*
DWFG						
Middle tertile	1.331 (0.633, 2.801)	0.451	0.960 (0.453, 2.034)	0.915	0.903 (0.419, 1.947)	0.794
Lowest tertile	4.992 (2.702, 9.226)	< 0.001	2.997 (1.586, 5.661)	0.001	2.574 (1.339, 4.950)	0.005
Infection-related death						
Middle tertile	0.383 (0.077, 1.902)	0.241	0.286 (0.057, 1.424)	0.126	0.261 (0.051, 1.330)	0.106
Lowest tertile	6.667 (2.767, 16.063)	< 0.001	4.257 (1.708, 10.608)	0.002	3.763 (1.481, 9.561)	0.005

The highest tertile was the reference group in all models.

Model 1, unadjusted model; model 2, adjusted for age, sex, and diabetes mellitus; model 3, adjusted for model 2 factors plus creatinine-cystatin C–based estimated glomerular filtration rate and serum albumin.

CI, confidence interval; DWFG, death with a functioning graft; HR, hazard ratio.

### Causes of death

Causes of patient death are shown in Table 5. Overall, infection was the most common cause of death (50%), followed by cardiovascular disease (30.8%) and malignancy (12.8%). In the lowest tertile, infection was the most common cause of death, accounting for 62% of all deaths. By contrast, cardiovascular disease was the most common cause of death in the middle plus highest tertiles (39.3%). In multivariable Cox analysis, the lowest creatinine-cystatin C ratio tertile was associated with an increased risk of infection-related death (aHR 3.763, 95% CI 1.481–9.561; *P* = 0.005) (Table 4, model 3).

**Table 5:** Causes of death according to creatinine-cystatin C ratio tertile.

Causes	Overall (n = 1592)	Lowest tertile (n = 530)	Middle + highest tertile (n = 1062)
Cardiovascular disease	24 (30.8%)	13 (26%)	11 (39.3%)
Infection	39 (50%)	31 (62%)	8 (28.6%)
Malignancy	10 (12.8%)	6 (12%)	4 (14.3%)
Trauma	2 (2.6%)	0	2 (7.1%)
Unknown	3 (3.8%)	0	3 (10.7%)
Total	78	50	28

Data are presented as number (percentage).

### Subgroup analysis

We performed subgroup analyses, stratified by recipient sex (male or female), age at creatinine-cystatin C measurement (< 55 or ≥ 55 y), BMI (< 23 or ≥ 23 kg/m^2^), creatinine-cystatin C–based eGFR (< 60 or ≥ 60 mL/min/1.73 m^2^), serum albumin level (< 4.0 or ≥ 4.0 mg/dL), and donor status (living or deceased donor). There were no significant interactions between subgroups and lowest creatinine-cystatin C tertile for DWFG, indicating that the association between a low creatinine-cystatin C ratio and an increased risk of DWFG persisted, regardless of recipient sex, age, BMI, creatinine-cystatin C–based eGFR, serum albumin, and donor status.

## Discussion

In this large single-center study, we investigated the clinical associations between sex-specific creatinine-cystatin C ratio and long-term outcomes after KT. We found that a low creatinine-cystatin C ratio was significantly associated with an increased risk of DWFG in KT recipients. Compared with the highest creatinine-cystatin C ratio group, the lowest creatinine-cystatin C ratio group had a 2.6-fold higher risk of DWFG, even after adjusting for potential confounding factors, including age, sex, diabetes mellitus, eGFR, and serum albumin. Infection was the most common cause of death in the lowest creatinine-cystatin C ratio group.

DWFG presents as distinctly different clinical phenotypes when compared with death-censored graft failure (the focus of most previous research and a condition caused by many different pathogenic processes)^8,16^. However, relatively few studies have examined risk factors for DWFG, despite DWFG accounting for approximately half of overall graft losses^9,17^. Infection, cardiovascular disease, and malignancy are common causes of DWFG. Recipient age, diabetes mellitus, and pre-transplantation duration of dialysis are known risk factors for DWFG, but these factors are not modifiable^18^. Little is known about post-transplantation factors predictive of DWFG. Only a limited number of studies have investigated the relationship between post-KT eGFR and DWFG, and the relationship remains unclear^8,18^. Therefore, clinical parameters that reliably identify patients at higher risk for DWFG are urgently required.

Significant graded associations between reduced eGFR and adverse outcomes, including cardiovascular events, hospitalization, and mortality, have been well established^19^. Although creatinine is the most frequently used biomarker for eGFR, creatinine-based eGFR has an “inverted J-shape” association with all-cause mortality^20,21^. By contrast, cystatin C has a strong and linear association with all-cause mortality^12^. Accumulating recent evidence suggests that the creatinine-cystatin C ratio may have utility as a surrogate marker for mortality beyond renal function^14,15^. Previous studies reported that a low creatinine-cystatin C ratio is associated with an increased risk of mortality in various conditions, such as diabetes mellitus^22^, chronic kidney disease^23,24^, malignancy^15^, and chronic obstructive pulmonary disease^25^. Our data demonstrated that a low sex-specific creatinine-cystatin C ratio was an independent risk factor for all-cause mortality in KT recipients. To our knowledge, this is the first study to evaluate clinical outcomes according to creatinine-cystatin C ratio in a large population of KT recipients.

One plausible explanation for the relationship between creatinine-cystatin C ratio and DWFG observed in the present study is that the ratio might reflect muscle mass. Since creatinine is an end-product of muscle catabolism, serum creatinine is affected by muscle mass^26,27^. In contrast, cystatin C is produced by all nucleated cells^28^. Therefore, a low creatinine-cystatin C ratio might be a marker of low muscle mass. In addition, the risk factors for low creatinine-cystatin C ratio found in our study were older age, longer dialysis duration, and re-transplant, which are all known risk factors for low muscle mass. Low muscle mass has emerged as a strong predictor of mortality in various populations. Several investigators, including our group, have reported an association between low muscle mass and poor transplant outcomes after KT^29,30^.

Another possible explanation for our findings is that cystatin C might reflect inflammatory status. Several studies have suggested that cystatin C is directly linked to adverse outcomes beyond renal function^11,13^, and previous studies reported that cystatin C is associated with inflammatory markers^31–33^. A low creatinine-cystatin C ratio, resulting from an elevated cystatin C level, may indicate an inflammatory state rather than a decline in renal function.

In addition, previous various studies have reported that patients with low muscle mass have increased vulnerability to infection and an increased risk of infection-related mortality^34–36^. Taking into account that the lowest tertile group might be associated with low muscle mass, this is consistent with our finding that infection is the most common cause of death in the lowest creatinine-cystatin C ratio group.

Our findings have potentially important clinical implications. Creatinine-based eGFR may overestimate GFR in patients with a low muscle mass, whereas cystatin C is less affected by muscle mass. Accordingly, the National Kidney Foundation (NKF) and the American Society of Nephrology have recommended the use of cystatin C to estimate GFR^37^. In situations where cystatin C and creatinine are measured concurrently, the creatinine-cystatin C ratio could be used as a potential clinical parameter for predicting DWFG, an outcome that is otherwise difficult to predict.

This study has several limitations. First, it was a retrospectively designed single-institution study, and the results of the study were not validated in an independent cohort. Therefore, further external validation of these study results is necessary. However, since it was conducted at a single center, the study has the advantages of consistent use of immunosuppressive drugs, standardized patient management protocols, and more complete and reliable data regarding mortality (including causes). Second, this study investigated cystatin C data measured at least 6 months after KT. It is therefore unknown whether the creatinine-cystatin C ratio has similar significance in predicting early mortality after KT. Third, because of the late introduction of cystatin C measurements, creatinine-cystatin C ratios were not obtained on the same day after KT across patients. In 2015, the NKF published guidelines recommending the use of cystatin C to evaluate kidney function post-KT, and cystatin C measurements were begun relatively recently in the Republic of Korea. Nevertheless, our data on the creatinine-cystatin C ratio have the advantage of representing real-world data. Fourth, since muscle mass was not measured, we were unable to directly assess the relationship between low muscle mass and creatinine-cystatin C ratio.

In conclusion, a low creatinine-cystatin C ratio was significantly associated with an increased risk of DWFG in KT recipients.

## Methods

### Patient selection

**Figure 4:**
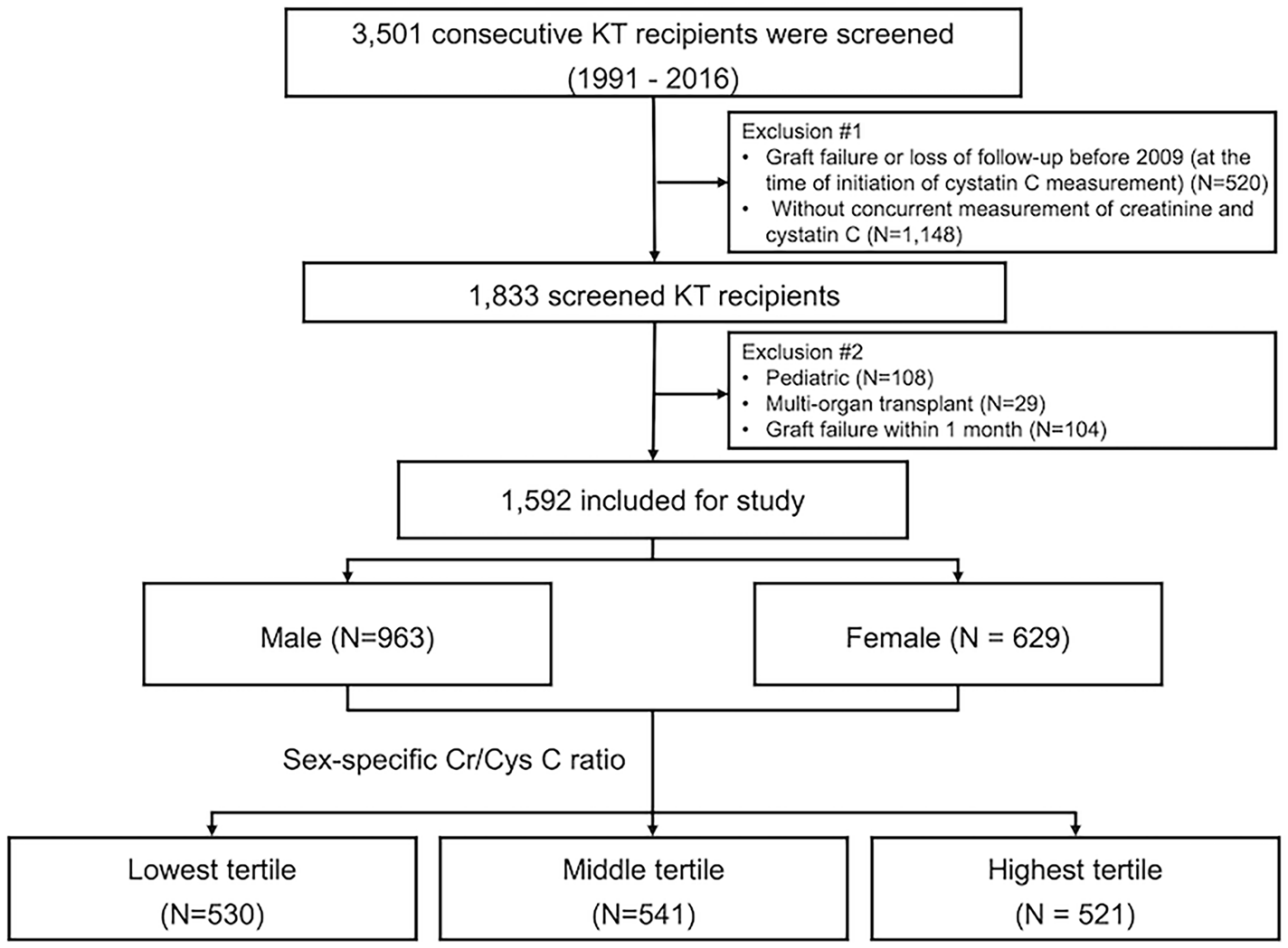
Flow diagram of the study. KT, kidney transplantation; Cr, creatinine; Cys C, cystatin C.

We screened consecutive adults who received a kidney transplant between January 1991 and December 2016 at Severance Hospital, Seoul, Republic of Korea. In this retrospective observational cohort study, we included patients with concurrent measurements of serum creatinine and cystatin C obtained at least 6 months after KT and excluded patients with graft failure or loss of follow-up before 2009 (at the time of initiation of cystatin C measurement). We also excluded patients who underwent multi-organ transplantation or experienced graft failure within 1 month after the creatinine-cystatin C ratio measurement. After excluding ineligible patients, 1592 KT recipients were included in this study. The patients were grouped into three groups based on sex-specific creatinine-cystatin C ratio tertiles (Fig. 4). The first creatinine (in mg/dL) to cystatin C (in mg/L) ratio at least 6 months after KT was used.

All study procedures were conducted in accordance with the Declaration of Helsinki and approved by the Institutional Review Board of Severance Hospital (4-2022-1560). The need for informed consent was waived owing to the retrospective study design.

### Data collection

Demographic and clinical data were retrieved from the Severance Kidney Transplant database. Laboratory and medication data were retrieved from the patients’ electronic medical records. Laboratory data included complete blood cell count (including hemoglobin), blood urea nitrogen, and serum levels of creatinine, cystatin C, albumin, calcium, total cholesterol, phosphorus, and uric acid. Serum creatinine was measured using the Jaffe kinetic method, and cystatin C was measured using the immunoturbidimetric assay. Estimated glomerular filtration rate (eGFR) was calculated using the recently published Chronic Kidney Disease Epidemiology Collaboration 2021 creatinine-cystatin C equation^5^.

### Immunosuppression and follow-up protocol

Maintenance immunosuppression consisted of a calcineurin inhibitor (tacrolimus or cyclosporine), prednisolone, and mycophenolate mofetil or a mammalian target of rapamycin inhibitor. All patients were followed at our transplant center. Patients visited the outpatient clinic every month for the first post-transplantation year and every 3 months thereafter. At each visit, history and physical examination were performed, and routine laboratory tests were obtained. At our institution, serum cystatin C levels have been measured annually in KT recipients at the physicians’ discretion since 2009.

### Clinical outcomes

The primary outcome of this study was DWFG. Secondary outcomes were cause of death and infection-related death. Patient survival was calculated from the date of creatinine-cystatin C ratio measurement to the date of death, loss to follow-up, or December 31, 2022 (end of the follow-up period). We also censored patients with graft failure (return to long-term dialysis or re-transplantation). Mortality data were obtained from both the electronic medical records of our transplant center and the Korean National Statistical Office, using a unique identifier.

### Statistical analyses

Data were expressed as frequency, mean ± standard deviation, or median and interquartile range (IQR), depending on the type of data. Chi-square test or Fisher’s exact test was used as appropriate to compare categorical variables. Continuous variables were compared using a one‐way analysis of variance for parametric data and the Kruskal–Wallis test for nonparametric data. Correlations between parameters were evaluated with the Pearson test. Multivariable logistic regression analysis was performed using the lowest creatinine-cystatin C ratio tertile as a binary outcome variable. Patient survival rates were analyzed using Kaplan–Meier curves and the log-rank test. Cox proportional hazard regression models were used to evaluate associations between creatinine-cystatin C ratio and study outcomes. Three models were created: model 1, unadjusted; model 2, adjusted for age, sex, and diabetes mellitus at the time of creatinine-cystatin C ratio measurement; and model 3, adjusted for the model 2 factors plus creatinine-cystatin C–based eGFR and serum albumin. A competing risk analysis for death with a functioning graft (DWFG) was conducted using the Fine and Gray subdistribution hazard model, with death-censored graft failure set as the competing risk. All tests were performed as two-tailed tests, and *P* values < 0.05 were considered statistically significant. Statistical analyses were performed using SPSS software (version 26.0; IBM Corp., Armonk, NY, USA) and R (version 3.6.3; R Foundation for Statistical Computing, Vienna, Austria).

### Supplementary Information

Supplementary Figures.

## Data Availability

The data that support the findings of this study are available from the corresponding author upon reasonable request.
