# Supplementary material for: Creatinine-cystatin C ratio and death with a functioning graft in kidney transplant recipients
Source: Sci Rep. 2024 Jan 23;14:1966. doi: 10.1038/s41598-024-52649-5 (PMC10806062; doi:10.1038/s41598-024-52649-5)
Supplement: Supplementary file 1 — Supplementary Figures. [file 41598_2024_52649_MOESM1_ESM.pdf]

Figure. S1. Correlation between serum creatinine and cystatin C levels.

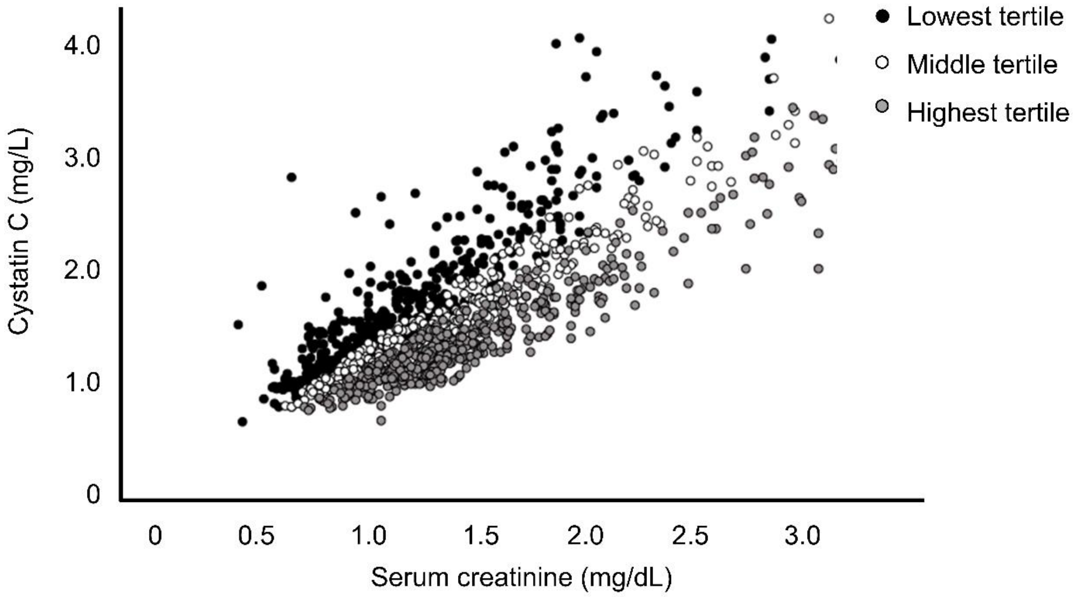

Figure. S2. Cumulative incidence of DWFG and DCGF between the lowest tertile and the middle+highest tertile groups.

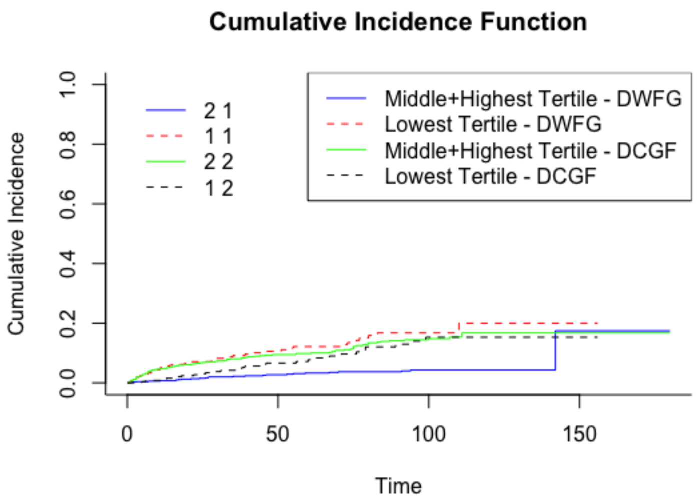

DWFG, Death with a functioning graft; DCGF, Death-censored graft failure; Time, months.
